# Cognitive impairment in clinically isolated syndrome: A systematic review

**DOI:** 10.1590/S1980-57642010DN40200002

**Published:** 2010

**Authors:** Carolina Fiorin Anhoque, Simone Cristina Aires Domingues, Antônio Lúcio Teixeira, Renan Barros Domingues

**Affiliations:** 1 Neuroscience Post-graduation Program, Federal University of Minas Gerais, Belo Horizonte MG, Brazil.; 2 Ph.D., Neuropsychologist, Vitória ES, Brazil.; 3 Professor of Neurology, Department of Internal Medicine, School of Medicine, Federal University of Minas Gerais, Belo Horizonte MG, Brazil.; 4 Professor, Department of Pathology, Santa Casa School of Health Sciences, Vitória ES, Brazil and Neuroscience Post-Graduation Program, Federal University of Minas Gerais, Belo Horizonte MG, Brazil.

**Keywords:** cognition, cognitive impairment, clinically isolated syndrome, demyelinating disease

## Abstract

The pattern of cognitive abnormalities in multiple sclerosis (MS) has been extensively studied and well characterized. However, little is known about the cognitive involvement in patients with the clinically isolated syndrome (CIS).

**Objectives:**

To perform a systematic review of the results of the studies on cognitive dysfunction in CIS patients.

**Methods:**

Cochrane, Lilacs, PubMed/Medline and SciELO databases were searched for studies involving patients with clinically isolated syndrome submitted to neuropsychological evaluation.

**Results:**

Seven studies fulfilled the selection criteria adopted in this review. The pattern of cognitive abnormalities in CIS resembles that found in patients with MS and is characterized by attention deficit, reduced information processing speed and impaired working memory and executive functions. The frequency of cognitive impairment in CIS seems to be lower than in MS.

**Conclusions:**

Cognition should be evaluated in patients with CIS. Future studies are required to evaluate the impact of cognitive abnormalities and to correlate them with axonal damage findings in patients with CIS.

## Abstract (pt)

As alterações cognitivas na esclerose múltipla (EM) são muito estudadas e bem caracterizadas. No entanto, pouco se sabe sobre o envolvimento cognitivo em pacientes com síndrome clínica isolada (SCI).

**Objetivos:**

Revisar sistematicamente resultados obtidos em estudos sobre cognição em pacientes com SCI.

**Métodos:**

Revisamos as seguintes bases de dados: Cochrane, Lilacs, Pubmed/Medline e Scielo buscando artigos em que pacientes com SCI foram submetidos à avaliação neuropsicológica.

**Resultados:**

Sete estudos preencheram critérios adotados pelos autores desta revisão. O padrão do prejuízo cognitivo em SCI é semelhante ao encontrado na EM, caracterizado por déficit de atenção, redução da velocidade de processamento, comprometimento da memória de trabalho e de funções executivas. A freqüência de disfunção cognitiva é inferior na SCI do que na EM.

**Conclusões:**

Pacientes com CIS devem ser avaliados quanto à cognição. Estudos futuros são necessários para avaliar o impacto do prejuízo cognitivo e correlacioná-lo com achados de dano axonal em pacientes com SCI.

Clinically isolated syndrome (CIS) is defined as the first episode of a demyelinating and inflammatory disease of the central nervous system (CNS). Several patients with CIS convert to multiple sclerosis (MS), a chronic demyelinating disorder characterized by CNS lesions disseminated in time and space.^1^ The most frequent CIS presentation is optic neuritis (ON). Only 22% patients with CIS without lesions on magnetic resonance imaging (MRI) convert to MS while 56% of those with MRI lesions will develop MS.^2^ Therefore, the risk of conversion to MS increases in CIS patients with MRI lesions in the CNS.

In recent years, evaluation of patients with MS has taken into account only motor and visual disability. However, recent studies have demonstrated that emotional, social, psychological and cognitive aspects have a tremendous impact on the quality of life of such patients.^3,4^ Consequently, the study of cognitive function in MS has gained great importance. Cognitive dysfunction in MS is found in 40 to 65% of patients^5^ and seems to be related with the number and localization of the demyelinating plaques, axonal loss, and brain atrophy typically found in MS.^6^ Considering that these pathological elements are progressive, it is important to establish in which phase of the disease the cognitive abnormalities began. Along these lines, some studies have assessed cognitive functions in early MS and CIS.^7-9^

In the present study a systematic review of the literature was conducted on cognitive dysfunction in patients with CIS, taking into account the neuropsychological tests used in these studies, the evaluation of potentially confounding variables such as depression and fatigue, and correlation with neuroimaging.

## Methods

The search strategy used the key words “clinically isolated syndrome”, “cognition”, and “cognitive impairment” to search the following database systems: Cochrane, Lilacs, Pubmed/Medline, and Scielo from January 1990 to December 2009.

Of the articles found, those which included patients with CIS characterized by the occurrence of a single attack of a clinical lesion suggestive of CNS demyelination and with MRI abnormalities, were selected. Articles that did not use a clear definition of ClS were excluded. Also, articles that did not present the cognitive assessment procedure were also excluded. The search was performed independently by three researchers.

A description of each study selected is first presented. Each study was analyzed according to the following pre-established parameters: study type, number of CIS patients included, inclusion of a control group, time between CIS and cognitive evaluation, percentage of CIS patients with cognitive impairment, main neuropsychological findings, correlation with imaging, and evaluation of psychiatric co-morbidities such as depression, anxiety, and fatigue.

## Results

Seventeen references were found, three in the Cochrane Library and fourteen on Pubmed/Medline. The search in the other databases retrieved no articles. Ten of the articles found were excluded for not meeting the inclusion and/or exclusion criteria established by the authors. The remaining 7 studies are presented below and in Table 1.

**Table 1:** Studies evaluating cognitive function in patients with clinically isolated syndrome (CIS).

Reference	Study type	Number of CIS patients	Control group	Time between CIS and cognitive evaluation	% of cognitive impaired CIS patients	Neuropsychological findings	Correlation between cognitive findings and neuroimaging	Psychiatric co-morbidities evaluation
Feinstein et al. 1992^4^	Cohort	16	No	4 and ½ years	Not available	Attention	Positive: T1 relaxation times	Yes (fatigue and depression)
Audoin et al. 2005^10^	Cross-sectional	18	18 controls	3 to 24 months	50	Processing speed	Positive: different pattern of functional MRI activation	No
Nilsson et al. 2008^12^	Cross-sectional	22	No	24-31 years	68 - 1 cognitive domain 31.8 - 2 or more cognitive domains	Visual spatial skillsExecutive functionsProcessing speed	Negative	No
Potagas et al. 2008^9^	Cross-sectional	33	43 controls	Not shown	27.3	Processing speedWorking memory	Not evaluated	Yes (depression)
Summers et al. 2008^13^	Cohort	25	No	7 years	48	AttentionProcessing speed	Positive: number of T2 lesions	Yes (anxiety and depression)
Feuillet et al. 2007^8^	Cross-sectional	40	30 controls	2.8 ± 0.5 years	80 - 1 cognitive domain 57.5 - two or more cognitive domais	Working and short term memoryProcessing speedAttention Executive functions	Not evaluated	Yes (depression)
Glanz et al. 2007^14^	Cross-sectional	15	27 controls	Not mentioned	49 - 1 cognitive domain 29 - 2 or more cognitive domains	Working memoryProcessing speedVerbal memory	Negative	Yes (depression)

The first published study included 48 patients with an isolated lesion of the type seen in MS.^4^ An initial cognitive evaluation was done after the first clinical manifestation and included Wechsler Adult Intelligent Scale (WAIS); Recognition memory tests for words and faces; Wisconsin card sorting test; speed of letter counting; auditory attention test; graded naming test; paired-associate learning test and the Story recall test. The authors found an attention decline but did not report the percentage of patients with this decline. After a 4.5-year follow up, 19 of the patients had converted to MS and 16 had not. Those patients who had converted to MS had greater visual memory impairment compared with those who had not converted. The attention scores remained unchanged, except in the group of MS patients classified as chronic progressive in whom the auditory attention tasks had deteriorated. The potential confounding effect of fatigue and psychiatric comorbidities was assessed in this study. The authors concluded that cognitive abnormalities can be seen in patients with an isolated lesion and that these abnormalities may worsen as conversion to MS occurs. In the MRI evaluation, T1 relaxation times in normal-appearing white matter correlated positively with cognitive impairment.

Audoin et al.^10^ compared 18 CIS patients with 18 healthy controls using the Paced Auditory Serial Attention Test (PASAT) and neuroimaging with functional MRI. The mean PASAT performance was lower in the CIS group. Half of the CIS patients had an impaired PASAT performance. These authors had found similar results in a previous small study^11^ involving 10 patients.

Nilsson et al.^12^ used a neuropsychological battery including the Boston Naming Test, 16-Item Token Test, Logical Memory, Rey Complex Figure, Trail Making Test, PASAT, and Wiscosin Card Sorting Test in 22 CIS patients aged 24 to 31 years after optic neuritis. Most (15 patients, 68%) had at least one affected cognitive domain and 7 had two or more domains affected. The most frequent affected domains were visual spatial skills, executive functions and processing speed. There was not a control group and psychiatric co-morbidities were not evaluated. No apparent correlation was found between MRI findings and cognitive functions.

Potagas et al.^9^ evaluated 33 CIS patients. These authors used the *Brief Repeatable Battery of Neuropsychological Tests* (BRBN) which includes Selective Reminding Test (SRT) - Long-term storage SRTL, consistent long-term SRTC and delayed recall SRTD; 10/36 Spatial Recall Test (SPART); Symbol Digit Modalities Test (SDMT), PASAT, and Word List Generation. Patients with impairment in three out of nine tests were considered as having cognitive impairment. The group also evaluated patients with relapsing remitting MS, secondary progressive MS, and healthy controls. The Beck Depression Inventory was used for depression screening. The percentage of cognitive impairment was 82.8% in patients with secondary progressive MS, 40% in the group of patients with relapsing remitting MS, and 27.3% in the CIS group. When comparing CIS and MS patients the authors found that CIS patients were less frequently impaired than patients with MS.

Summers et al.^13^ evaluated 62 patients, seven years after presenting CIS. The neuropsychological evaluation included the Wechsler Adult Intelligence Scale-Revised, story and figure recall subtests of the Adult Memory and Information Battery, Spatial Working Memory - CANTAB and Hayling Sentence Completion Task, Brixton Spatial Anticipation Test, PASAT, and Symbol Digit Modalities Test (SDMT). Those falling below the 5^th^ percentile or < 2 SD below the mean were considered to have impairment. There was no control group and psychiatric co-morbidities were not evaluated. At the time of evaluation, 25 patients were still classified as having CIS, 12 of whom were cognitively impaired and 13 cognitively unimpaired. The most frequent cognitive impairment was attention/speed of information processing. There was a correlation between the number of T2 lesions 3 months after CIS episode and attention impairment.

Feuillet et al.^8^ evaluated 40 CIS patients with a mean of 2.8±0.5 years of disease. The authors used the Brief Repeatable Battery of Neuropsychological Tests (BRBN). Thirty healthy controls were also evaluated. The Montgomery and Asberg Depression Rating Scale was used to assess depressive symptoms. A total of 80% of the CIS patients and 50% of controls obtained scores below the normal range on at least one test. Twenty three (57%) CIS patients and two controls (7%) failed at least two tests (p<0.0001). The most commonly affected cognitive abilities were working memory and short term memory, speed of information processing, attention, and executive functions. There was no correlation between depression and cognitive findings.

Glanz et al.^14^ evaluated 92 patients with CIS or newly diagnosed MS. Fifteen patients were classified as having CIS although the authors did not evaluate the findings of CIS and MS patients separately. Twenty seven healthy controls were also evaluated. The Brief Repeatable Battery of Neuropsychological Tests (BRBN) was used. Depression was evaluated with the Center for Epidemiological Studies Depression Scale (CES-D). Fourty nine CIS/MS patients demonstrated impairment in at least one cognitive measure and 20% were impaired on at least two measures. The most common impairment was found in working memory and processing speed followed by verbal memory. No correlation was observed between depression and the cognitive deficits or between cognitive findings and MRI lesions.

In these seven studies, a total of one hundred sixty nine CIS patients were cognitively evaluated. Most of these studies were cross-sectional. The time of neuropsychological assessment varied from three months to 31 years after CIS onset. The percentage of cognitively impaired CIS patients ranged from 27% to 80% of the patients according to the neuropsychological battery and the normality threshold adopted in each study. The neuropsychological tests also varied considerably from study to study. Nevertheless, the pattern of affected cognitive domains was similar across the studies. The most affected neuropsychological domains were attention, processing speed, working memory and executive functions. Association between cognitive dysfunction and neuroimaging finding was determined in four out of five studies. Most of the studies evaluated a potential confounding effect of depression on cognitive functioning, but none found any association between depression and neuropsychological impairment. However, other psychiatric disorders and fatigue were not investigated.

## Discussion

The cognitive domains principally affected in MS are memory, processing speed, attention, and executive functions. Fully developed dementia is rare in MS patients but the mild cognitive impairment may result in significant functional disability.^15,16^ According to the CIS selected studies cited above, the pattern of cognitive impairment found in CIS patients is the same as previously described for MS patients.^5,9^ Therefore, the cognitive findings in MS may appear from the first demyelinating attack or even before. However, little is known about how these cognitive abnormalities change with disease evolution. In two of the above-mentioned studies the cognitive findings of CIS patients were compared with cognitive findings of MS patients. Feinstein et al.^4^ found that attention deficit persisted 4 and ½ years after the first attack but memory declined in the group of patients who converted to MS yet not among those who still had CIS after that time. Potagas et al.^9^ found that cognitive disturbances were more frequent in MS patients than in CIS patients. These studies suggest that cognitive abnormalities are slightly more frequent and more pronounced in MS than in CIS patients. This issue is very important since several studies have demonstrated that the early introduction of immunomodulatory medications, such as interferons and glatiramer acetate, can delay the conversion of CIS to MS.^15-18^ Future studies should investigate whether the use of such medications in CIS patients influence the outcome of cognitive function.

There is not yet a clear explanation of why cognitive impairment occurs in CIS. The mechanisms of cognitive abnormalities in CIS are most likely the same as those in MS. The mechanisms that lead to the development of cognitive abnormalities in patients with multiple sclerosis have been studied. It has been shown that the burden of T-2 weighted lesions of some cerebral areas is associated with attention and working memory. This suggests that cognitive performance is affected by the number and the localization of demyelinating CNS lesions, affecting the functioning of brain circuits involved in these cognitive functions.^19^ Studies have also shown that cognitive deterioration increases in parallel with the decrease in brain parenchymal volume.^20^ Since brain atrophy is a marker of the neurodegenerative process in MS, this suggests that axonal loss is one of the determinants of cognitive impairment in MS.^21^ Considering that besides symptomatic lesions, patients with CIS have silent demyelinating lesions,^3^ it is possible that these lesions may interfere with the functioning of brain circuits related with cognition. Also, it has been demonstrated that axonal degeneration begins in early stages of MS and CIS.^3^ Thus, early neurodegeneration may be another explanation as to why these cognitive abnormalities are evident in patients with CIS. Further studies should analyze the precise role of the localization of brain lesions, the burden of white matter lesions, and the extent of axonal damage in the cognitive performance of CIS patients.

We conclude that there is sufficient evidence that cognitive impairment is present in CIS patients, resembling the cognitive findings of MS patients, albeit less frequent and less intense. Future studies are necessary to assess the impact of these abnormalities on quality of life of CIS patients. A more detailed investigation of the influence of psychiatric co-morbidities on the cognitive pattern of CIS patients is also warranted. Future studies should also address whether patients with CIS and distinct clinical presentations have different patterns of cognitive performance. The future studies in this field should take into account the high heterogeneity of this syndrome and therefore should include more homogeneous CIS populations in terms of time elapsed between clinical onset and neuropsychological evaluation, and with regard to clinical presentation. Cognitive impairment is highly disabling in MS patients^3,5^ given that it can be found when patients first present with CIS, the inclusion of neuropsychological evaluation in clinical and therapeutic studies will allow a better understanding of the initial stages of this disease.
